# Pinch-Off Formation in Monolayer and Multilayers MoS_2_ Field-Effect Transistors

**DOI:** 10.3390/nano9060882

**Published:** 2019-06-14

**Authors:** Yonatan Vaknin, Ronen Dagan, Yossi Rosenwaks

**Affiliations:** School of Electrical Engineering, Tel-Aviv University, Tel-Aviv 69978, Israel; ronendagan@gmail.com (R.D.); yossir@tauex.tau.ac.il (Y.R.)

**Keywords:** 2D materials, KPFM, MoS_2_, pinch-off

## Abstract

The discovery of layered materials, including transition metal dichalcogenides (TMD), gives rise to a variety of novel nanoelectronic devices, including fast switching field-effect transistors (FET), assembled heterostructures, flexible electronics, etc. Molybdenum disulfide (MoS_2_), a transition metal dichalcogenides semiconductor, is considered an auspicious candidate for the post-silicon era due to its outstanding chemical and thermal stability. We present a Kelvin probe force microscopy (KPFM) study of a MoS_2_ FET device, showing direct evidence for pinch-off formation in the channel by in situ monitoring of the electrostatic potential distribution along the conducting channel of the transistor. In addition, we present a systematic comparison between a monolayer MoS_2_ FET and a few-layer MoS_2_ FET regarding gating effects, electric field distribution, depletion region, and pinch-off formation in such devices.

## 1. Introduction

Two-dimensional (2D) materials, such as graphene and transition metal dichalcogenides (TMDs), attract extensive interest from the research community as they are considered as possible candidates for post-silicon electronics [1]. Molybdenum disulfide (MoS_2_), is considered a promising candidate for various applications in microelectronics [2,3,4,5,6], optoelectronics [7,8], sensing [9,10], spintronics [11,12], and many others [13,14] due to its unique electrical properties [15,16] and thermal stability. The performance of the applications listed above is entangled with specific electrostatic behavior at the interface between the MoS_2_ and the other compounds within the device [17].

Depletion region formation at MoS_2_ homojunctions, heterojunctions, and metal junctions has been modeled and investigated by many groups [17,18,19,20]. Nipane et al. [21] modeled the electrostatics of lateral junctions in atomically thin materials using line charges representation, Sohn et al. [22] investigated the electrostatic band alignment at Au/MoS_2_ contacts as a function of the thickness of MoS_2_ exfoliated flakes, and Chiu et al. [23] determined the band alignment in single-layer MoS_2_/WSe_2_ heterojunctions using micro X-ray photoelectron spectroscopy and scanning tunneling microscopy (STM).

Kelvin probe force microscopy (KPFM) is a powerful tool for the direct measurement of the surface potential of semiconductors [24]. Several groups have used this technique to monitor the work function difference between the different number of layers of MoS_2_ samples [25], studying the effect of the substrate on the electrostatic properties of MoS_2_ layers [26], and assessing the effect of gas and molecular adsorption on chemical vapor deposition (CVD)-grown MoS_2_ flakes [27,28,29]. Other groups have measured the built-in potential of single-layer MoS_2_ heterojunctions using KPFM [30] and demonstrated the electrical properties of the contact between MoS_2_ and different metals [31]. These measurements resemble the use of KPFM for contact resistance evaluation [32] and contact-free mobility estimation [33] in thin-film organic transistors.

Current saturation in thin-film FET devices is attributed to both velocity saturation and pinch-off formation [6,34]—moreover, it is a major accelerator of the device’s performance, controlling the output conductance and on/off ratio of the transistor [3]. However, no experimental results on the pinch-off effect in ultrathin TMDs have been published. In this work we present a method for the direct observation and determination of the pinch-off voltage of a thin-layer TMD FET through the measurement of the electrostatic potential along the conducting channel of the transistor in operando. We observe, for the first time to our knowledge, pinch-off formation in such devices and discuss the differences between single- and few-layer MoS_2_ FET devices.

## 2. Materials and Methods

Monolayer and few-layer MoS_2_ samples were transferred on top of an 8 mm square dye made of a highly doped P-type silicon wafer covered by a 90 nm silicon oxide (SiO_2_) layer via the mechanical exfoliation with scotch tape, initially developed for graphene [35], of MoS_2_ crystals supplied by Structure Probe Inc. (SPI) Supplies (West Chester, PA, USA). The wafer was patterned with gold alignment marks prior to exfoliation using optical lithography, and monolayer and few-layer MoS_2_ flakes were identified by their contrast using an optical microscope. Contacts made of 50 nm gold on top of a 3 nm titanium were designed by E-beam lithography and evaporated using an electron-beam evaporator (VST, Israel), and lift off was conducted using N-Methyl-2-pyrrolidone (NMP) at 80 °C. The devices were then placed on a chip carrier, wire bonded, transferred into a N2 glove box and annealed at 95 °C to reduce humidity.

The electrical measurements and device characteristics, performed inside a N2 glove box, were conducted using a semiconductor parameter analyzer (B1500A, Agilent Technologies, Santa Clara, CA, USA) and atomic force microscope (AFM)-based amplitude modulation-Kelvin probe force microscopy (AM-KPFM) (Bruker, MA, USA). The measuring step size was ~20 nm, enabling in situ measurements of the electrostatic potential with lateral resolution approaching the limit of the KPFM in nitrogen atmosphere [36].

## 3. Results and Discussion

**Figure 1:**
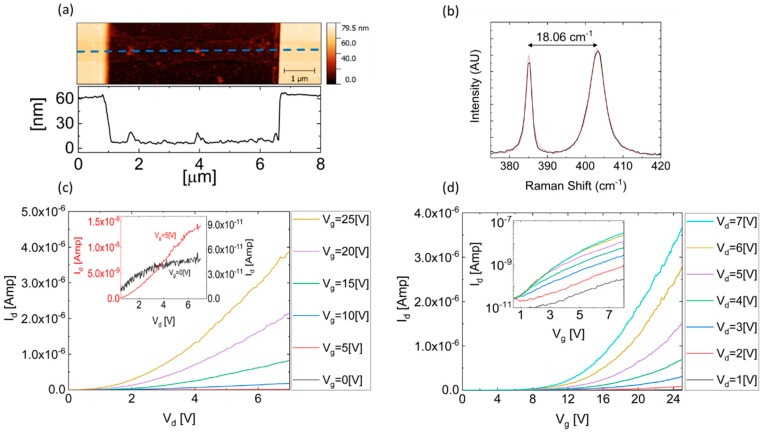
(**a**) An atomic force microscope (AFM) image, in addition to a topography profile along the dashed blue line, of the single-layer molybdenum disulfide (MoS_2_) device. (**b**) Raman spectrum of the single-layer MoS_2_ flake, presenting a separation of 18.06 cm^−1^ between $E_{2g}^{1}$ and $A_{1g}$. (**c**) $I_{d}\left(V_{d}\right)$ characteristics of the single-layer MoS_2_ field-effect transistor (FET) device for several gate voltages, in addition to the magnified image in the inset, presenting the $I_{d}\left(V_{d}\right)$ curves for $V_{g}=0\;V$ and $V_{g}=5\;V$ in blue and red, respectively. (**d**) $I_{d}\left(V_{g}\right)$ characteristics of the FET device for several drain voltages, in both linear scale and logarithmic scale in the inset.

An AFM image of a monolayer MoS_2_ FET with a channel length (L) of 5.5 um and a width (W) of 0.75 um, in addition to a topography profile along the device, are shown in Figure 1a. Raman spectroscopy, presenting a separation of 18.06 cm^−1^ between $E_{2g}^{1}$ and $A_{1g}$ corresponding to a single layer of MoS_2_ [37], is presented in Figure 1b. Figure 1c shows the $I_{d}\left(V_{d}\right)$ characteristics of the device for several gate voltages ($V_{g}$), while Figure 1d depicts the $I_{d}\left(V_{g}\right)$ curves in linear scale for several drain voltages ($V_{d}$) showing n-type transistor behavior, as expected from unintentionally doped exfoliated MoS_2_ flakes [38,39,40]. Logarithmic scale $I_{d}\left(V_{g}\right)$ curves, for a gate voltage range of 0.5 to 8 V in which the high drain voltage curves are united into a single curve and separated as the gate voltage increases, as expected in the presence of pinch-off, are presented in the inset. Standard metal–oxide–semiconductor field-effect transistor (MOSFET) theory [41] was used to extract the field-effect mobility from the $I_{d}\left(V_{g}\right)$ curves in the linear regime, and found to be 2.52 cm^2^/V∙s. Current saturation, as depicted in the inset of Figure 1c for gate voltages of 0 and 5 V, is likely to take place in FET devices, being more possible evidence of pinch-off formation near the drain electrode.

**Figure 2:**
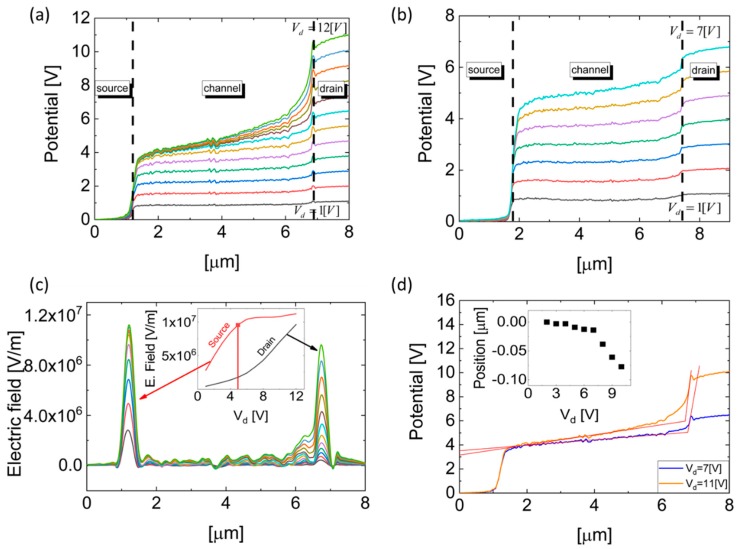
(**a**) Electrostatic potential measurements at varying drain voltages ($V_{d}=1\;V$ to $V_{d}=12\;V$ with 1 V steps), while gate and source electrodes are grounded. (**b**) Electrostatic potential measurements at varying drain voltages ($V_{d}=1\;V$ to $V_{d}=7\;V$ with 1 V steps), while $V_{g}=5\;V$ and the source electrode is grounded. (**c**) Electric field distribution along the transistor corresponding to the bias conditions in (a). The inset shows the electric field at the Au/MoS_2_ interface near the source and drain electrodes as a function of the drain voltage at $V_{g}=0\;V$. (**d**) Pinch-off position extraction at $V_{g}=0\;V$ for representative drain voltages. In the caption, the pinch-off evaluation as a function of the drain voltage is given.

The electrostatic potential distribution along a single-layer MoS_2_ FET device, as a function of the biased electrodes—source, drain, and gate, measured in situ using KPFM—is presented in Figure 2. Figure 2a shows the electrostatic potential along the conducting channel for several drain voltages where the gate is grounded. It is observed that a depletion region, resulting in an increased electrostatic potential slope near the drain electrode, starts to form at a drain voltage of 3 V, while starting at $V_{d}=6\;V$ the separation between the different electrostatic potential curves becomes narrower and the potential drop near the drain electrode becomes steeper. This abrupt voltage drop near the drain electrode is attributed to pinch-off formation near the electrode. Figure 2b presents the electrostatic potential along the device with $V_{g}=5\;V$. In this case, due to the positive gate voltage and the corresponding electrostatic field, electrons from the different electrodes are injected into the conducting channel, and a larger drain voltage (of around 6 V) is consequently required for the formation of the depletion region.

Following the Schottky–Mott theory [42,43], the barrier height in the metal–MoS_2_ interface is determined by the difference between the work function of the metal and the electron affinity of the MoS_2_. Following this model, assuming $V_{d}=V_{s}=0\;V$, the built-in potential, and consequently the depletion region, between the gold electrodes and the channel is determined by:

(1) $$\psi_{bi}=\phi_{Au}-\left(\chi_{MoS_{2}}+\frac{\left(E_{c}-E_{f}\right)}{q}\right)+V$$

where $\phi_{Au}$ is the gold work function, $\chi_{MoS_{2}}$ is the MoS_2_ affinity, $E_{c}-E_{f}$ is the energy difference between the conduction band and the Fermi level of the MoS_2_, and V is the potential induced by the gate electrode. Assuming a gold work function of 5.4 eV [44], a MoS_2_ electron affinity of 4 eV [45], and an unintentionally n-type doping concentration of $1.4\times 10^{12}\left[\frac{1}{cm^{-2}}\right]$ [38] shifting the Fermi level position of the MoS_2_ towards the conduction band, a Schottky barrier height of 1.4 eV will be formed and a built-in potential resulting in a depletion region will be created. Increasing the gate voltage will increase the charge carrier concentration in the conducting channel, and the depletion region will become narrower.

The charge concentration induced by the gate is:

(2) $$Q=C_{OX}\left(V_{gs}-V_{t}-V\left(x\right)\right)$$

where $C_{OX}$ is the structure capacitance per unit area, $V_{t}$ is the threshold voltage, and V(x) is the measured electrostatic potential minus the electrostatic potential at $V_{gs}=0\;V$ [46]. Following this equation, a point with no charge will be created near the drain when $V\left(x\right)=V_{drain}=V_{gs}-V_{t}$; this is commonly defined as the pinch-off. Any additional increase in the drain voltage will increase the pinch-off region, inducing a larger electric field in this region, as depicted in Figure 2c above. By increasing the gate voltage, a large concentration of carriers is injected into the channel, and the pinch-off voltage is increased accordingly.

Figure 2c shows the electric field distribution across the single-layer MoS_2_ FET device for drain voltages between 1 and 12 V, for $V_{gs}=0\;V$. The electric field is calculated as the first derivative of the electrostatic potential distribution, measured by the KPFM, as presented in Figure 2a. The inset of Figure 2c presents the electric field at the source–MoS_2_ and MoS_2_–drain interfaces as a function of increasing $V_{ds}$. At drain voltages lower than the pinch-off voltage, the electric field is accumulated at the source–MoS_2_ interface, while at some other voltages, namely the pinch-off voltage, the electric field at the source–MoS_2_ interface remains constant and the electric field at the MoS_2_–drain interface increases. A vertical line marking the drain voltage at which the electric field becomes $1\times 10^{6}\;V/m$, representing the pinch-off voltage, is also shown in Figure 2c. Given a gate voltage of 0 V, the threshold voltage can be calculated using the following formulation: $V_{t}=-V_{pinch-off}\approx -5\;V$.

As the electric field at the MoS_2_–drain interface increases, the portion of depleted channel required to screen this field increases. In order to establish a rigorous method for pinch-off positioning, we determined the pinch-off point in a manner similar to how the slope transition is extracted in logic device state determination. Following this method, the pinch-off position is defined as the intersection between the linear fit of the plateau region of the electrostatic potential along the conducting channel and the linear fit of the steepest electrostatic potential slope at the MoS_2_–drain interface. The results are presented in Figure 2d, while the inset of Figure 2d shows the pinch-off position as a function of the drain voltage. As expected, the pinch-off position remains constant for drain voltages lower than the pinch-off voltage and moves towards the source as the drain voltage increases beyond this voltage.

**Figure 3:**
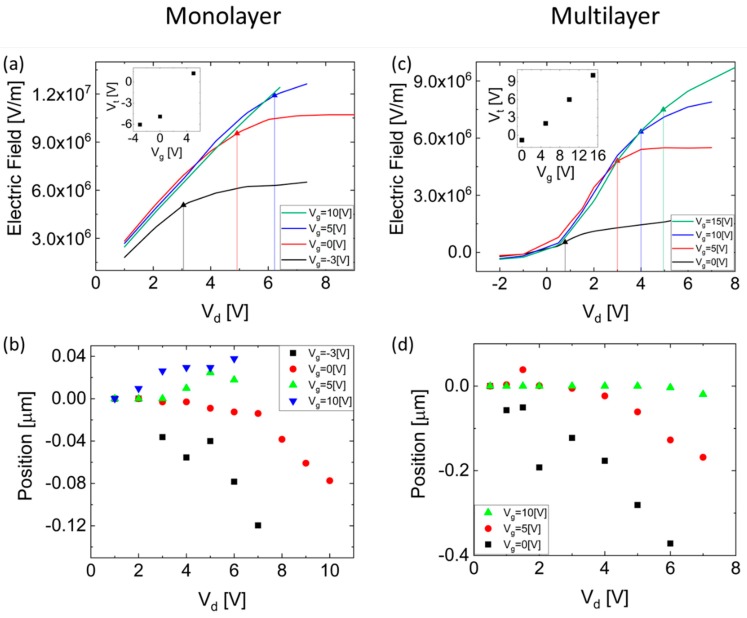
(**a**) The electric field at the Au–MoS_2_ interface near the source electrode as a function of the drain voltage for varying gate voltages; vertical lines representing the pinch-off voltages for the different bias conditions are also presented. The inset shows the threshold voltage extracted for the different gate voltages in a single-layer MoS_2_ transistor. (**b**) Pinch-off position as a function of the drain voltage extracted for varying gate voltages in a single-layer MoS_2_ transistor. (**c**,**d**) are similar to (**a**,**b**), respectively, for a few-layer MoS_2_ FET.

Figure 3 shows a detailed comparison between single-layer and multilayer (composed of 4–5 layers and exhibiting 20 cm^2^/V∙s field-effect mobility) MoS_2_ FET devices. Figure 3a shows the accumulated electric field at the source–MoS_2_ interface as a function of the drain voltage for several gate voltages for the monolayer MoS_2_ FET device; the vertical lines represent the pinch-off voltage as described above. The threshold voltage, extracted using the above formula as a function of the gate voltage, is shown in the inset. Figure 3b demonstrates the pinch-off widening at the MoS_2_–drain interface as a function of the drain voltage for several gate voltages for the monolayer device, where the zero position is the pinch-off location for $V_{d}=0\;V$ and the negative values represent pinch-off widening into the conducting channel away from the drain. As expected, an increased gate voltage, resulting in enhanced conducting channel, requires a higher drain voltage to achieve pinch-off.

Figure 3c,d show the accumulated electric field at the source–MoS_2_ interface, in addition to the corresponding threshold voltages, and demonstrate the pinch-off widening at the MoS_2_–drain interface for a multilayer MoS_2_ FET device. As shown, the corresponding pinch-off voltages in the multilayer device are smaller relative to the monolayer device. This decrease in the pinch-off voltage with an increasing number of layers may arise from the unintentional doping in the exfoliated MoS_2_ samples, which is known to be attributed to sulfur vacancies [39,40,47,48,49] formed mostly at interfaces, resulting in a higher carrier concentration in monolayer samples compared to multilayers [50]; this larger carrier concentration increases the threshold and pinch-off voltages.

Sulfur vacancies in addition to intentional doping of thin-film FETs will cause variations in device performance including on/off ratio, mobility, and current saturation. Observation of the pinch-off phenomenon, extraction of the pinch-off voltage, and calculation of the corresponding threshold voltage enables us to distinguish between the governing mechanisms in such devices. In addition, since the mobility in such devices is known to be field dependent [34] at drain voltages higher than the pinch-off voltage, the electric field along the device will remain constant, and hence the mobility will be independent of the drain bias. We have presented good agreement between the measured pinch-off voltage (Figure 3a) and the current saturation voltage presented in Figure 1c. Consequently, we deduce that the pinch-off phenomenon is the dominant mechanism of current saturation in the measured devices, and that the device performance is not limited by carrier velocity saturation.

## 4. Conclusions

In summary, a KPFM-based study was conducted to investigate the pinch-off phenomenon in thin-layer MoS_2_ FET devices. We presented a direct observation of pinch-off region formation in monolayer and multilayer MoS_2_ FETs through a detailed analysis of the electrostatic potential distribution along the devices. We showed the pinch-off dependence, in terms of both pinch-off voltage and widening of the pinch-off region, on the applied bias and derived the threshold voltage accordingly. It was shown that the pinch-off voltage decreased with the increase of the charge carrier concentration within the conducting channel, which is consistent with the increased sulfur vacancies of MoS_2_ surfaces compared to multilayers. Better understanding of the pinch-off phenomenon, in addition to its crucial effect on device performance, in few–layer materials is a key point for designing the next generation of TMD-based devices.
